# Comparative Assessment of the Inhibitory Potential of the Herbicide Glyphosate and Its Structural Analogs on RGD-Specific Integrins Using Enzyme-Linked Immunosorbent Assays

**DOI:** 10.3390/ijms232012425

**Published:** 2022-10-17

**Authors:** Borbála Gémes, Eszter Takács, Inna Székács, Robert Horvath, András Székács

**Affiliations:** 1 Agro-Environmental Research Centre, Institute of Environmental Sciences, Hungarian University of Agriculture and Life Sciences, Herman O. út 15, H-1022 Budapest, Hungary; 2 Nanobiosensorics Laboratory, Institute of Technical Physics and Materials Science, Centre for Energy Research, Konkoly-Thege M. u. 29-33, H-1121 Budapest, Hungary

**Keywords:** integrins αVβ3, α5β1, αllbβ3, glyphosate, structural analogs, AMPA, metabolites, SARS-CoV-2 receptor binding domain, enzyme-linked immunosorbent assay, ELISA, inhibition

## Abstract

Transmembrane glycoprotein integrins play crucial roles in biochemical processes, and by their inhibition or activation, different signal pathways can be disrupted, leading to abnormal physiological functions. We have previously demonstrated the inhibitory effect of glyphosate herbicide’s active ingredient on cell adhesion and its αvβ3 integrin antagonist effect. Therefore, it appeared particularly exciting to investigate inhibition of glyphosate and its metabolites on a wider range of Arg-Gly-Asp (RGD) binding integrins, namely αvβ3, α5β1 and αllbβ3. Thus, the purpose of this study was to assess how extended the inhibitory effect observed for glyphosate on the integrin αvβ3 is in terms of other RGD integrins and other structurally or metabolically related derivatives of glyphosate. Five different experimental setups using enzyme-linked immunosorbent assays were applied: (i) αvβ3 binding to a synthetic polymer containing RGD; (ii) αvβ3 binding to its extracellular matrix (ECM) protein, vitronectin; (iii) α5β1 binding to the above polymer containing RGD; (iv) αllbβ3 binding to its ECM protein, fibrinogen and (v) αvβ3 binding to the SARS-CoV-2 spike protein receptor binding domain. Total inhibition of αvβ3 binding to RGD was detected for glyphosate and its main metabolite, aminomethylphosphonic acid (AMPA), as well as for acetylglycine on α5β1 binding to RGD.

## 1. Introduction

Integrins are bidirectional signaling receptors that play important roles in development, cell–cell interactions, and pathological processes. As heterodimers, transmembrane receptors contain two subunits (α and β). The ligand-binding sites specifically recognizing ligands from the extracellular matrix (ECM) are located in the external head segments of both subunits [1]. At the internal side of the cell membrane, the cytoplasmic tail of the integrins connects to the cytoskeleton, initiating the signaling cascades which make chemical and mechanical signal transduction possible [2]. Depending on their various structures, integrins can bind different ECM proteins (one or more, sometimes in overlap with each other) [3] and mediate a number of crucial cellular physiological processes, such as survival, proliferation, motility and differentiation [4]. Various types of the subunits exist; for example, the 18 α and 8 β subunits are known in humans, forming 24 members [5] of the four integrin families, i.e., integrins binding leukocytes, collagen, laminin or the Arg-Gly-Asp (RGD) tripeptide [6]. Upon ligand binding, integrins undergo conformational rearrangements that initiate various internal cascades in the cell as a response to the external stimulus (outside-in signaling) [7].

**Figure 1:**
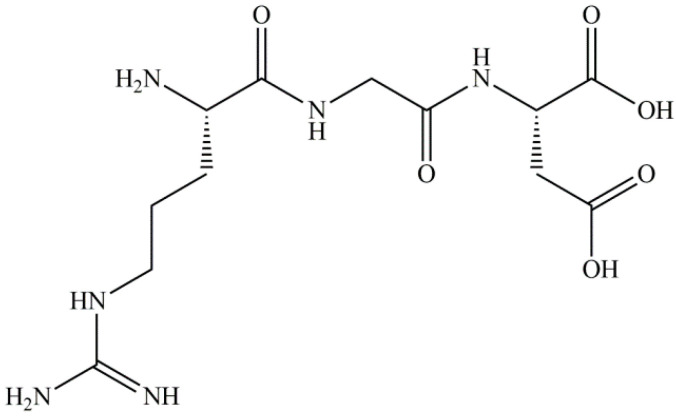
Chemical structure of the Arg-Gly-Asp (RGD) tripeptide sequence.

In 1984, the RGD tripeptide sequence (Figure 1) was described by Pierschbacher and Ruoslahti as the shortest binding motif which can be recognized by 8 of the 24 human integrins, namely α5β1, α8β1, αvβ1, αvβ3, αvβ5, αvβ6, αvβ8 and αllbβ3 [1,8]. Many ECM proteins contain this sequence, e.g., fibrinogen, fibronectin, vitronectin, the latency-associated proteins of TGF-β1 and TGF-β3, or nephronectin [1]. Although all members of the family recognize the RGD tripeptide by having its receptor in their β subunit, other residues on the α subunit of the integrin are also considered to contribute to ligand-specificity and affinity [9]. On the basis of their broad spectrum of biochemical roles in cell adhesion, proliferation, migration and angiogenesis, RGD-specific integrins became a popular target in the diagnostics, imaging and drug therapy studies of several diseases, including fibrotic diseases, rheumatoid arthritis and thrombosis [10,11,12]. In the last decade, RGD-containing ligands have been investigated as potential tools against cancerous diseases. The overexpression of certain integrins has been observed in several cancer types. Thus, the overexpression of the αvβ3 integrin receptor has been associated with various types of cancer including melanoma, glioblastoma, breast, prostate, pancreatic, ovarian, cervical and colon cancer [13].

Some human pathogenic bacteria, fungi and viruses use integrins to attach to target cells. *Staphylococcus aureus* Clumping Factor A binds fibrinogen and the von Willebrand factor, and the major cell wall protein PspC of *Streptococcus pneumoniae* binds vitronectin to crosslink the bacteria to the integrin αvβ3 [14,15]. The outer membrane protein A of *Escherichia coli* contains the RGD motif and binds directly to αvβ3. Several viruses appear to utilize their expression of the RGD motif to facilitate their attachment to or entry into host cells via interactions with the host cell’s integrins. Clinically important RGD-containing viruses include the human metapneumovirus, human adenovirus subtype 5, human cytomegalovirus and the Epstein–Barr virus [16]. A recent actuality of the RGD motif in the COVID-19 pandemic is that after Wei et al. determined the ACE2 receptor-binding domain of the SARS-CoV-2 spike protein (S-protein) in 2020 [17], Sigrist and colleagues highlighted the presence of the RGD sequence on the surface of the protein not far from the ACE2 binding region and suggested its potential role in the entry into the host cell [18].

Integrins also participate in several processes, the disruption of which may lead to the occurrence of non-transmittable diseases. Various xenobiotics are known to disturb cellular processes through receptors [19], including integrins. Integrin’s antagonists are mostly oligopeptides or larger peptides [20,21], which are often promising for therapeutic purposes [22]. Occasionally, small ligands can also behave as allosteric integrin inhibitors [23,24,25,26,27] and are also frequently used as pharmaceuticals. A recent example of a small, non-peptide xenobiotic found to exert a unique and specific integrin-targeting activity is a phosphonomethylamino acid derivative, glyphosate [*N*-(phosphonomethyl)glycine] [28]. Originally, this compound became known not as a pharmaceutical but as the world’s leading pesticide [29,30]. In the more than 50 years since its introduction for weed control, this compound became the most often and most widely used active ingredient of herbicides today. It is an organophosphorous substance with high water solubility, and its phytotoxicity relies on its unique inhibitory activity of the enzyme 5-enolpyruvyl-3-shikimate phosphate synthase (EPSPS), a key biocatalyst in the biosynthesis of aromatic amino acids (phenylalanine, tyrosine, tryptophan) in plants [31,32]. Beside its pre-emergent application against perennial weeds, this non-selective broad-spectrum active ingredient of herbicides is also used as a desiccant prior to harvest [33], and its agricultural use has been boosted since the introduction and large-scale cultivation of genetically modified glyphosate-tolerant crops [30]. The drastically increasing application rates have resulted in rising incidents and levels of soil, water and food contamination with the parent compound or its decomposition products, primarily the main metabolite aminomethylphosphonic acid (AMPA). Importantly, glyphosate has not only become a ubiquitous surface water contaminant [30,34,35] but it has also been found in livestock and in human urine and blood [30,36]. As an active ingredient, glyphosate is up for re-registration in the European Union in December 2022. Prior to its previous re-authorization in 2017, it was classified as a Group 2A carcinogen (probably carcinogenic to humans) by the International Agency for Research on Cancer [37,38], which was refuted by the European Food Safety Authority, the European Chemicals Agency and other regulatory agencies [39,40]. As a result, in its last re-registration in 2017, its authorization was extended only for 5 years [30] (not for 10 years, as in the usual case), and the issue of carcinogenicity has remained controversial and under strong societal debate to date, as illustrated by the diversity of opinions on the topic [41,42].

Our previous study revealed the inhibitory effect of glyphosate on αvβ3 integrin’s binding to the RGD sequence using a label-free biosensor method and a modified enzyme linked immunosorbent assay (ELISA). Using these techniques, we revealed almost full inhibition at 11 mM [28]. In the present study, further glyphosate-related compounds were investigated in the ELISA format, both on the above integrin and on two other RGD-binding integrins, extending the scope of the assessment to broader ranges of integrin proteins and small ligands with structural similarities to glyphosate. Thus, AMPA and *N*-acetylglyphosate, as the main metabolites of glyphosate, and glycine, acetylglycine (aceturic acid), sarcosine and iminodiacetic acid (as minor metabolites and amino acid analogs) were studied, along with glyphosate.

## 2. Results and Discussion

Whether the antagonistic effect of glyphosate occurs with other integrins, and whether it is attributable only to glyphosate or also to some of its derivatives (metabolites and the accompanying impurities) were our key questions to be explored. It was also intriguing to find out which molecular features may play a role in the newly recognized effect of glyphosate, and studying the corresponding activities of the structurally related analogs promised to shed light on this question.

**Figure 2:**
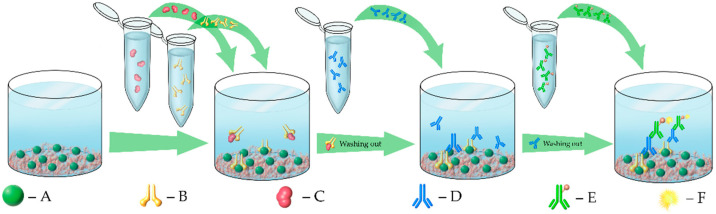
Schematic illustration of the enzyme-linked immunosorbent assay (ELISA). The wall of each well of a 96-well microplate was coated with a macromolecule showing high affinity to a given integrin (an RGD sequence-based synthetic polymer or ECM protein, e.g., vitronectin, fibrinogen or S-protein (the SARS-CoV-2 spike protein receptor binding domain)) and the remaining free surface was blocked with bovine serum albumin (BSA) (**A**). The corresponding integrin (**B**) was allowed to interact with the surface-immobilized macromolecules in the presence or absence of the ligands (glyphosate and its related compounds) at various concentrations ranging from 0 to 22 mM (**C**) that could modulate this binding process. The integrin molecules bound to the coating macromolecules were detected by an integrin-specific primary antibody (**D**), followed by an IgG-specific secondary antibody conjugated to a tracer enzyme (horseradish peroxidase, HRP) (**E**) and a colorimetric reaction of HRP using hydrogen peroxide as a substrate and 3,3′,5,5′-tetramethlybenzidine (TMB) as a chromophore (**F**). If the ligand inhibited binding of the integrin molecules to the coating macromolecules, further steps of the ELISA process were blocked, leading to no color signal formation.

Therefore, the integrin-binding affinity immunoassays were expanded beyond αVβ3 to other related integrins, namely α5β1 and αllbβ3. To assess the binding capacities, a phase-heterologous competitive affinity-binding immunoassay (Figure 2) was used. All three integrins chosen in this study are members of the RGD family.

**Figure 3:**
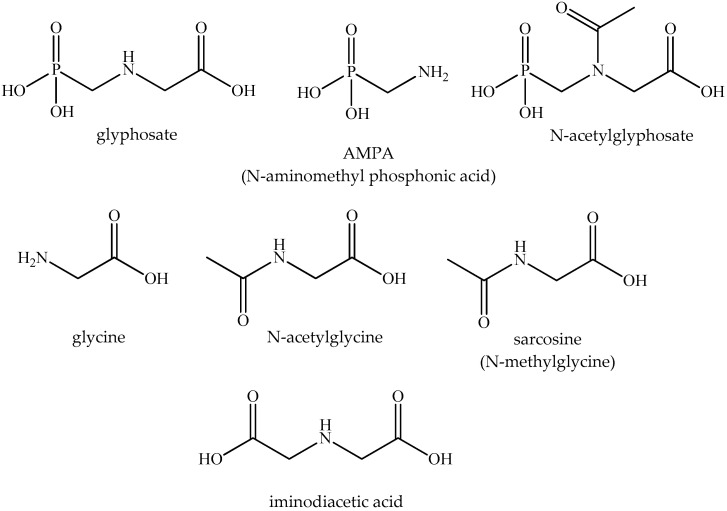
Chemical structures of the ligands (the active herbicide ingredient glyphosate and its structural analogs) investigated in this study.

The ligands tested (Figure 3) included the target compound, glyphosate, in the form of its isoprolylammonium salt, which is the form that occurs in numerous glyphosate-based herbicide formulations including Roundup Classic, and several of its common or less frequent metabolites or contaminating substances (Table 1). Thus, AMPA is a degradation product of glyphosate formed via the degradation of phosphonate microbially in the soil or via photodegradation in water [43], *N*-acetylglyphosate is a major metabolite in glyphosate-tolerant crops genetically modified to express the transgenic enzyme glyphosate *N*-acetyltransferase [44], and the other compounds tested are minor metabolites, product impurities or substances of comparative relevance. As seen from their physicochemical parameters, they are all water-soluble, with the highest aqueous solubility shown by glyphosate itself, a very unique and uncommon feature among pesticides’ active ingredients. These features are all well-reflected in their characteristics of lipophilicity (the octanol–water partition coefficients) (Table 1).

All test configurations were set according to our previously developed assay on the effect of glyphosate on the RGD-binding capability of αVβ3 integrin, where almost full (96.3 ± 3.2%) inhibition was detected at 11 mM [28,47]. In this study, the highest concentration (22 mM, 5.02 g/L) investigated was 1.4 times higher than the concentration of glyphosate in the herbicide formulation Roundup Classic at the 1:100 dilution (3.6 g/L) recommended by the manufacturer in agricultural applications. Thus, 22 mM is slightly higher than the environmentally relevant concentration of glyphosate. Glyphosate and the seven related compounds were studied in the concentration range of 0.1–22 mM, and their inhibitory effects on the binding of (1) αVβ3 to its ECM protein vitronectin, (2) αVβ3 to RGD sequence, (3) α5β1 to RGD sequence and (4) αllbβ3 to its ECM protein (fibrinogen) were investigated. The inhibitory effect of glyphosate on (5) the binding of αVβ3 to S-protein, the SARS-CoV-2 spike protein receptor binding domain, was also tested. The inhibitory activities of glyphosate and the structurally related ligands tested in Cases (1)–(4) are shown in Table 2.

### 2.1. Inhibitory Effects on the Integrin αVβ3

The inhibitory effect of glyphosate and its structural analogs on αVβ3 ranged from ~25 to 100%, depending on the surface affinity binding agent and the ligand. Generally, it can be concluded that glyphosate and its metabolite, AMPA, caused total and almost total inhibition, respectively, of the RGD-displaying polymer, while in the ECM protein-based system, their effect decreased. For glyphosate, the lowest inhibitory effect was obtained for binding the S-protein integrin. In contrast, the opposite effects were detected for the structural analogs, i.e., their inhibitory effects were higher for the RGD-displaying system than in the ECM protein-based assay.

#### 2.1.1. Using a Synthetic Polymer Containing the RGD Motif as A Surface Affinity Binding Agent

The application of the synthetic polymer displaying the RGD motif showed that RGD was the only binding site on the plate’s surface. As a result, it can be considered that both glyphosate and AMPA cause total inhibition at a concentration of 22 mM (100.0 ± 3.9% and 95.9 ± 2.2% for glyphosate and AMPA, respectively) on αVβ3. The IC_50_ values (concentration at 50% inhibition) were also not significantly different (2.7 ± 0.5 mM and 1.3 ± 0.2 mM for glyphosate and AMPA, respectively). The structural analogs also exerted an inhibitory effect; however, they were below the 50% inhibition level even at the highest concentration used (Table 2).

#### 2.1.2. Using Vitronectin as a Surface Affinity Binding Agent

Vitronectin is one of the ECM proteins of αVβ3. In the ECM-based ELISA assay, both glyphosate and AMPA caused lower inhibition than in the RGD-based tests. The inhibition levels were 44.8 ± 3.6% and 75.3 ± 2.1% for glyphosate and AMPA, respectively. In contrast, the inhibition of *N*-acetylglyphosate (47.5 ± 1.2%), acetylglycine (58.5 ± 0.8%) and iminodiacetic acid (52.4 ± 4.2%) increased compared with the RGD-based system. For glycine and sarcosine, the inhibitory effect was not significantly different between the RGD-based and ECM-based assays (Table 2).

#### 2.1.3. Using S-Protein, the SARS-CoV-2 Spike Protein Receptor Binding Domain, as a Surface Affinity Binding Agent

The spike protein of the SARS-CoV-2 virus contains the RGD motif; thus, the inhibitory effect of glyphosate on the binding of the spike protein and αVβ3 was also investigated. Glyphosate inhibited the binding of αVβ3 by 35.6 ± 4.4%. This result indirectly shows that the S protein of SARS-CoV-2 can exert its effect through RGD binding and also through other biochemical routes. As glyphosate had the strongest effect on αVβ3, and its effect was far the strongest among the ligands, only this integrin and only glyphosate were investigated in the current study with the SARS-CoV-2 S-protein.

### 2.2. Inhibitory Effects on the Integrin α5β1

All ligands had an inhibitory effect on the binding of α5β1 to the RGD sequence, but total inhibition was achieved by acetylglycine (98.9 ± 2.3%). In the RGD-based ELISA assay, the inhibitory effects of glyphosate and AMPA were 3.7 times and more than 17.5 times lower, respectively, compared with αVβ3. Other ligands (*N*-acetylglyphosate, glycine, sarcosine and iminodiacetic acid) exerted 8–22% inhibition (Table 2).

### 2.3. Inhibitory Effects on the Integrin αllbβ3

Of all the ligands, only glyphosate inhibited the binding of the integrin αllbβ3 to fibrinogen by 40.2 ± 2.6% at a 22 mM concentration. No other compounds had inhibitory effect. Moreover, AMPA and acetylglycine had activation on binding of integrin αllbβ3 to fibrinogen (Table 2).

**Figure 4:**
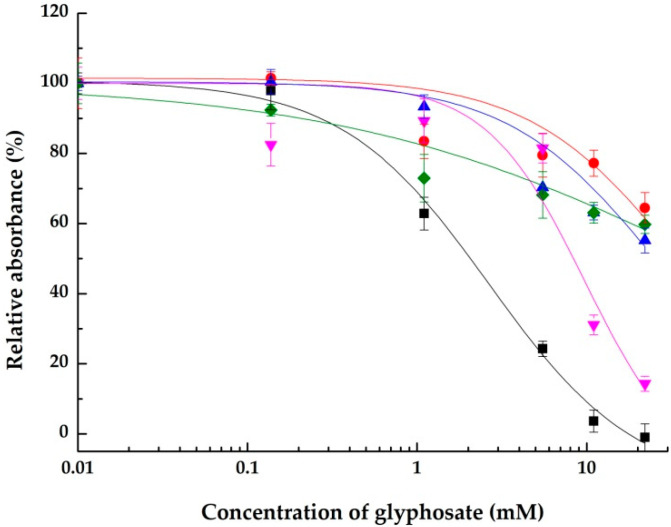
Concentration-dependent inhibitory effects of glyphosate on the integrins αVβ3 using RGD (black), α5β1 using RGD (pink), αVβ3 using vitronectin (blue), αllbβ3 using fibrinogen (green) and αVβ3 using S-protein (the SARS-CoV-2 spike protein receptor binding domain) (red). Data are shown as means ± SD.

**Figure 5:**
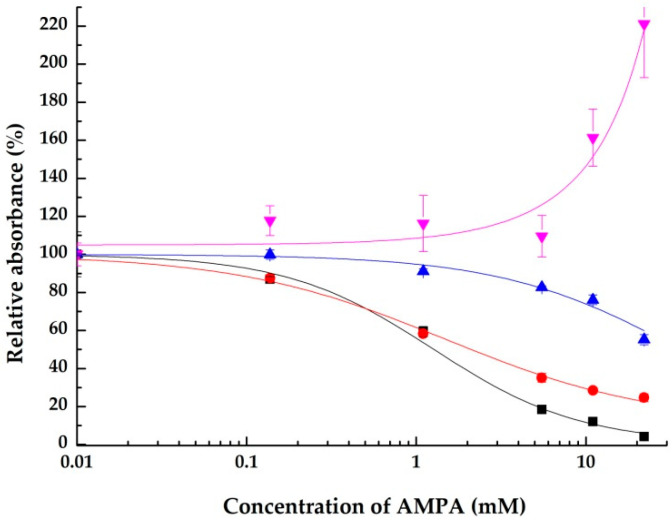
Concentration-dependent inhibitory effects of AMPA on the integrins αVβ3 using RGD (black), α5β1 using RGD (blue), αVβ3 using vitronectin (red) and αllbβ3 using fibrinogen (pink). Data are shown as means ± SD.

The concentration-dependent effects of glyphosate and AMPA on all the integrins tested are summarized in semilogartihmic plots in Figure 4 and Figure 5, respectively.

For glyphosate, it can be concluded that the active ingredient of herbicides inhibits the RGD binding part of the integrins. In RGD-based assays, where a synthetic polymer containing RGD was immobilized onto the surface of ELISA microplate, for the αVβ3 and α5β1 integrins, 100.0 ± 3.9% and 85.7 ± 2.1% inhibition was detected, respectively. In ECM protein- or possibly S protein-based assays, where besides RGD motif, other binding parts of the proteins were presented to the integrins, the maximum inhibition was 44.8 ± 3.6% in the assay of αVβ3 using vitronectin (Figure 4). The high affinity of glyphosate to the RGD binding part of integrins was evident, since among the ligands tested, glyphosate was the only one that inhibited the connection of αllbβ3 and its ECM protein, fibrinogen. The structure of integrins contains a divalent binding site that is near or at the ligand-binding site and is necessary for the formation of the ligand–integrin connection. The hypothesis is that the carboxy terminus of glyphosate mimics the aspartic acid in the RGD motif, where cation exchange between the glyphosate molecule and the integrin can be realized, and the glycine in glyphosate can form a hydrophobic bond with the integrin molecule [20,28]. In our study, glycine exerted a lower inhibitory effect of 39.5 ± 6.6% and 22.1 ± 3.3% in RGD-based ELISA systems than glyphosate. For the full inhibition of glyphosate, it is supposed that both the carboxy terminus and the glycine part of glyphosate are necessary for binding to the integrin.

For the main glyphosate metabolite, AMPA, it was determined that this ligand also showed high affinity for the RGD binding site of integrins; however, this affinity was lower than that of glyphosate. Moreover, the activation of the αllbβ3 integrin in binding to fibrinogen was also determined. For αVβ3, at a 22 mM concentration, 95.9 ± 2.2% and 75.3 ± 2.1% inhibition was detected in the RGD-based and ECM-based assays, respectively.

**Figure 6:**
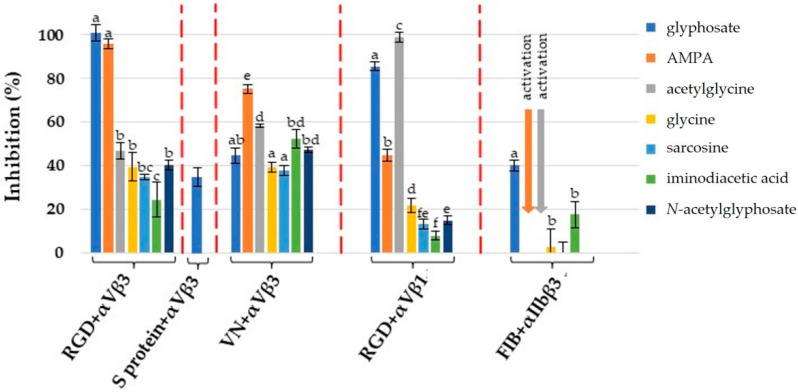
Maximal inhibitory potential of glyphosate and its structurally related compounds. Data are shown as means ± SD. Red dashed lines separate the different ELISA systems (RGD-based, ECM protein-based and SARS-CoV-2 S-protein-based). Small letters above the columns represent the statistical differences between the separate ELISA systems.

Complete inhibition of certain integrins was achieved only by glyphosate, AMPA and acetylglycine. Nonetheless, the inhibitory potential at the highest concentration applied, 22 mM (depicted on Figure 6), is also informative for comparing the binding affinity of the given ligands to different integrins.

Amplifying the activity of integrin αllbβ3 by AMPA and acetylglycine appears to be unique within the observations of the present study. Previous reports have indicated that the low-affinity state of integrins is stabilized by disulfide bonds between the Cys moieties in the coiled-coil structure of integrins (e.g., αllbβ3), and that small molecule reagents (e.g., the reducing agent dithiothreitol) can interfere with these disulfide bonds and thus, activate αllbβ3 [48,49]. Acetylglycine, however, is not a reducing agent; therefore, its effect does not appear to be related to the known activation process of disulfide bonds.

### 2.4. Biochemical and Possible Medical Relevance

The abovementioned integrins αvβ3 and α5β1 are mainly expressed in endothelial cells, lymphocytes, macrophages, thrombocytes and osteoblasts, but αvβ3 is also expressed in neutrophil cells, smooth muscle tissue, osteoclasts and fibroblasts, whereas αllbβ3 is mainly expressed in megakaryocytes and thrombocytes [50]. Since the biochemical role of integrins in organisms is complex, a description of all their roles was not the aim of this study. Our previous survey [28] was the first to report the identified inhibitory effect of glyphosate on cell adhesion processes involving the integrin αvβ3. The results obtained in a biosensoric measurement setup clearly demonstrated that glyphosate significantly reduced the adhesion of MC3T3-E1 cells coated with the RGD motif via blocking RGD-specific integrins in a concentration-dependent manner. This implies that all physiological processes that involve such cell adhesion can potentially be affected when exposed to glyphosate above a concentration of 8.7%. To highlight the importance of any inhibition of activation of these transmembrane receptors, here, we mention certain physiological processes in which they play a notable role. The integrin αvβ3 is highly expressed in activated endothelial cells, newborn vessels and some tumor cells, and takes part in processes of angiogenesis such as tumor growth. It regulates tumor cells’ adhesion and migration, and thus the process of metastasis [51]. However, this integrin is not present in resting endothelial cells and most normal organ systems, making it a suitable target for anti-angiogenic therapy [52]. The SARS-CoV-2 spike protein receptor binding domain (SARS-CoV-2 S protein) interacts with αVβ3 via the RGD motif and causes events of vascular leakage; thus, integrins’ interactions can explain several COVID-19-induced events of endothelial dysfunction [53]. Glyphosate is an efficient inhibitor if RGD is the obligate binding site. In presence of further recognizing sequences, its effect becomes weaker, indicating, as circumstantial evidence, that the SARS-CoV-2 S-protein does not only bind to αvβ3 via the RGD motif. The adhesion receptor αVβ3 integrin regulates macrophage differentiation and macrophages’ responses to external signaling, and its activation can maintain chronic inflammatory processes in pathological conditions [54].

The integrin α5β1, as an endothelial cell integrin, is indispensable in vascular development and maturation. The subunit α5 of integrins have critical roles in blood vessel development during embryogenesis and the complexity of overall blood vessel patterns [55]. Lethal vasculature and cardiac defects were detected when a gene encoding the α5 subunit was knocked out [56]. Furthermore, α5β1 is a major functional integrin expressed on the surface of undifferentiated mouse embryonic stem cells, and the expression and function of this integrin are critical steps in stem cells’ differentiation [57].

The integrin αIIbβ3 is highly expressed in platelets and their progenitors, and thus it has a central role in platelet function, hemostasis and arterial thrombosis [58]. Its activation promotes the processes of generating thrombin and blood coagulation [58]; therefore, it is a target in anti-thrombosis therapy. Paradoxically, however, several αIIbβ3 antagonists mimicking the RGD motif have been developed in the hope of preventing thrombosis, which resulted in increasing the likelihood of its incidence due to their integrin-activating capability [59].

All these integrins are known to bind to echistatin [60,61], while αllbβ3 is inhibited, unlike the others, by tirofiban [23] and epifibatide [62]. The SARS-CoV-2 S-protein investigation proves their relevance not only to non-transmittable diseases but also to viral pathogens, as seen in the literature [14,15,16,17,18].

Altogether, the specific physiological consequences of the inhibitory potency of glyphosate on integrins is hard to predict. At this stage, making concrete predictions would be highly speculative, and caution regarding not making conclusions that are not substantiated by scientific evidence in the area of its possible health effects has been urged in the scientific literature [41]. Thus, concerning the possible health effects, more systematic studies are needed with the involvement of at least animal models. The results could also lead to more specific integrin-targeting compounds or specific surface coatings facilitating cell adhesion.

## 3. Materials and Methods

### 3.1. Chemicals

All chemicals, including the salts for the buffers, fibrinogen, vitronectin, SARS-CoV-2 S-protein, anti-mouse immunoglobulin conjugated to horseradish peroxidase (HRP) as a secondary antibody and the inhibitors were purchased from Merck Life Science Kft. (Budapest, Hungary) and its legal predecessors, unless stated otherwise. Glyphosate (*N*-phosphonomethyl glycine, CAS No: 1071-83-6) was used in the tests in form of its isopropylammonium salt (CAS No: 386411-94-0) obtained from Toronto Research Chemicals (Toronto, ON, Canada), along with its derivative, *N*-acetylglyphosate (CAS No: 129660-96-4). The physicochemical characteristics of the ligands used in the current study are listed in Table 1. The integrins αvβ3 and α5β1 were purchased from R&D Systems Inc. (Minneapolis, MN, USA), whereas αllbβ3 was obtained from Enzyme Research Laboratories Ltd. (Swansea, UK). The primary antibodies (CD51/CD61, CD49e and CD41b) were purchased from Becton, Dickinson and Company (Franklin Lakes, NJ, USA).

### 3.2. Enzyme-Linked Immunosorbent Assays (ELISAs)

Assays were carried out in high-capacity 96-well microplates (Nunc, Roskilde, DK, #442404). Plates were coated with 100 µL of 250 μg/mL PLL-g-PEG-RGD in the form of poly(L-lysine)-graft-poly(ethylene glycol) terminated with the sequence GGGGYGRGDSP (SuSos, Dübendorf, Switzerland) in 10 mM 4-(2-hydroxyethyl)-1-piperazine ethanesulfonic acid (HEPES, pH = 7.4) for 1 h at room temperature or the ECM proteins vitronectin (0.5 μg/mL) or fibrinogen (20 μg/mL), both in a carbonate buffer (15 mM Na_2_CO_3_, 35 mM NaHCO_3_, pH = 9.6) overnight at 4 °C. Coatings with SARS-CoV-2 S-protein were made with a 5.72 μg/mL solution in pure PBS (137 mM NaCl, 2.7 mM KCl, 10 mM Na_2_HPO_4_ × 2H_2_O, 3 × 250 µL/well, pH = 7.4) for 4 days at 4 °C. The plates were washed three times with PBS and 0.1% Tween 20. The wells were blocked for 1 h at room temperature with 150 µL 1% bovine serum albumin in a Tris buffer, referred to hereafter a TrisB (20 mM Tris-HCl, 150 mM NaCl, 1 mM CaCl_2_, 1 mM MgCl_2_ and 1 mM MnCl_2_; pH = 7.5). After washing, competition was performed by incubating 50 µL/well of the inhibitor, which was first added in serial dilution in the 137.5 µM–44 mM range (in TrisB), together with 50 µL/well of integrin (αVβ3 at 2 μg/mL, αVβ3 in the SARS-CoV-2 S-protein test at 4 μg/mL, α5β1 at 2.5 μg/mL and αllbβ3 at 5 μg/mL in TrisB). After 1 h of incubation at room temperature and three rounds of washing, 100 μL/well of the primary antibody (mouse anti-human CD51/61, CD51/61 in SARS-CoV-2 S-protein test, CD49e or CD41b at concentrations of 2, 5, 1 and 4 μg/mL in TrisB) was added. Incubation and three rounds of washing were followed by adding the secondary antibody anti-mouse IgG-HRP at a volume of 100 μL/well (1 μg/mL, TrisB) and incubation for 1 h at room temperature. The wells were washed three times, then 100 μL/well of 1.2 mM of hydrogen peroxide as a substrate of HRP was added with 1.2 mM of chromophore 3,3′,5,5′-tetramethylbenzidine in a 0.5 M citrate buffer (pH 5.0). Color-forming enzymatic reactions were stopped after the required time with 50 μL/well of 4 N sulfuric acid, and the wells were read at 450 nm using a SpectraMax iD3 Multi-Mode Microplate Reader (Molecular Devices, San Jose, CA, USA) in endpoint mode.

### 3.3. Statistical Analysis

The effects of the glyphosate-related compounds on integrins’ binding to their ligands were statistically analyzed by R 4.0 (The R Foundation for Statistical Computing, Vienna, Austria). Shapiro–Wilk and Levene’s tests were used to check the normality of the data and the homogeneity of the variances, respectively, in each assay. Normality in the Shapiro–Wilk tests and variance homongeneity in Levene’s tests were accepted if *p* > 0.05. Statistical evaluation of the data was performed by using a general linear model. Tukey’s honest significant difference (HSD) tests were conducted as post hoc analyses following ANOVA to assess the significant differences between groups. IC_50_ values were visualized in semi-logarithmic plots and were calculated by non-linear regression using a logistic (5-parameter) sigmoid dose–response equation described by Rodbard [63], with *p* values ≤ 0.05 considered statistically significant. Visualization of the semi-logarithmic curves was performed by the OriginLab OriginPro 7.0 data analysis and graphing software system (OriginLab Corporation, Northampton, MA, USA).

The results are reported as means ± standard deviation (SD). Mean values were calculated as the average of the replicates. In case of independent treatments, replicates were considered individually, i.e., the mean values were determined as the average of the replicates in all treatments (not as the average of the averages from each treatment).

## 4. Conclusions

This study was devised to assess how extended the inhibitory effect of the active herbicide ingredient glyphosate on the integrin αvβ3, identified in our previous report [28], is in terms of other RGD integrins and other structurally or metabolically related derivatives. On the basis of the results of the inhibition tests carried out in phase heterologous integrin receptor binding immunoassay formats, we reached several conclusions. Full inhibition, similar to what was previously identified, and complete blocking of the binding of αvβ3 to the coating macromolecule by glyphosate was detectable only in certain cases: glyphosate and AMPA inhibited αvβ3 from binding to RGD, and acetylglycine also inhibited α5β1 from binding to a synthetic polymer containing the RGD motif. Thus, inhibition by glyphosate appears to be specific for αvβ3, which has a key role in angiogenic processes, e.g., in tumor growth. Only partial inhibition (8.0–58.5%) was achieved with certain glyphosate metabolites, while others showed negligible inhibitory potency.

Beside almost full inhibition of αvβ3′s binding to RGD (95.9 ± 2.2%) and high inhibition of αvβ3′s binding to vitronectin (75.3 ± 2.1%), AMPA activated the binding of αIIbB3 to its ECM protein, fibronectin, possibly due to its Ca^2+^-binding capability. The same pattern was detected for acetylglycine. Although inhibition levels of 46.8 ± 3.7%, 58.5 ± 0.8% and 98.9 ± 2.3% were detected for the integrins αvβ3 with RGD, αvβ3 with the ECM protein vitronectin and α5β1 with RGD, respectively, for αIIbB3, high activation was determined.

## Figures and Tables

**Table 1:** Physicochemical properties of the ligands studied.

Compound	CAS No. ^1^	Molecular Mass	Water Solubility (g/L)	Clog P_o/w_ ^2^	Comment
Glyphosate Glyphosate isopropylammonium salt ^3^	1071-83-6 386411-94-0	169.07 228.19	157 (pH 7.0) 1050	−4.6 * −4.16	Active ingredient of herbicides
Aminomethylphosphonic acid (AMPA)	1066-51-9	111.04	56 (20 °C)	−4.7 *	Metabolite
*N*-acetylglyphosate	129660-96-4	211.11	slight	−2.4 *	Metabolite
Glycine	56-40-6	75.07	250 (25 °C)	−3.2 *	Amino acid, metabolite
Acetylglycine	543-24-8	117.10	26.3 (15 °C)	−1.2 *	Acylamino acid
Sarcosine	107-97-1	89.09	89.1 (20 °C)	−3.2 −2.8 *	Metabolite
Iminodiacetic acid	142-73-4	133.10	24.3 (5 °C)	−3.3 *	Minor impurity

^1^ The Chemical Abstract Service registry number is an individual identifier of the chemicals. ^2^ The logarithm of the partition coefficient of the compound between a non-polar organic phase (octanol) and water, as a descriptor of lipophilicity. Values marked with * were calculated by the XLOGP3-AA atom-additive method in the PubChem public database of the National Library of Medicine (Bethesda, MD, USA) [45,46]. ^3^ Shown as supplementary information. The isopropylammonium salt of glyphosate is used in certain glyphosate-based herbicide formulations to increase the water solubility of the active ingredient. The physicochemical properties of glyphosate and its isopropylammonium salt are different; therefore, both entities are listed for clarity.

**Table 2:** IC_50_ values and maximal inhibition (%) of the ligands (glyphosate and its structural analogs) investigated on integrins.

ELISA	IC_50_ Value (mM) ^1^ (Maximal Inhibition Achieved)			
**Integrin**	αVβ3	αVβ3	α5β1	αllbβ3
**Immobilized affinity reagent**	RGD	Vitronectin	RGD	Fibrinogen
**Compounds**				
Glyphosate	**2.7 ± 0.5** (100.0 ± 3.9%)	>22 (44.8 ± 3.6%)	**9.7 ± 3.2** (85.7 ± 2.1%)	>22 (40.2 ± 2.6%)
Aminomethylphosphonic acid (AMPA)	**1.3 ± 0.2** (95.9 ± 2.2%)	**1.5 ± 0.4** (75.3 ± 2.1%)	>22 (44.8 ± 2.6%)	>22 (activation) ^2^
*N*-acetylglyphosate	>22 (40.6 ± 2.2%)	>22 (47.5 ± 1.2%)	>22 (15.0 ± 2.0%)	>22 (n. d. ^3^)
Glycine	>22 (39.5 ± 6.6%)	>22 (39.5 ± 2.4%)	>22 (22.1 ± 3.3%)	>22 (n. d.)
Acetylglycine	>22 (46.8 ± 3.7%)	**9.2 ± 1.0** (58.5 ± 0.8%)	**5.8 ± 0.9** (98.9 ± 2.3%)	>22 (activation)
Sarcosine	>22 (35.0 ± 1.2%)	>22 (37.9 ± 2.3%)	>22 (13.4 ± 2.1%)	>22 (n. d.)
Iminodiacetic acid	>22 (24.6 ± 7.9%)	**11.5 ± 1.5** (52.4 ± 4.2%)	>22 (8.0 ± 2.0%)	>22 (n. d.)

^1^ The IC_50_ value refers to the concentration at which the substance studied exerted half of its maximal inhibitory effect in the ELISA assay. ^2^ Activation means that instead of inhibition, an enhanced effect was determined, which resulted in a higher absorbance value than the negative control. ^3^ Not detected.

## Data Availability

The data presented in this study are available on request from the corresponding author. The data are not publicly available for privacy reasons.
